# The Fun Factor: Does Serious Gaming Affect the Volume of Voluntary Laparoscopic Skills Training?

**DOI:** 10.1007/s00268-020-05800-y

**Published:** 2020-09-28

**Authors:** Wouter Martijn IJgosse, Harry van Goor, Camiel Rosman, Jan-Maarten Luursema

**Affiliations:** 1 Department of Surgery, Radboud Institute for Health Sciences, Radboud University Medical Center, Geert Grooteplein Zuid 10, 6525 GA Nijmegen, The Netherlands; 2 Radboud University Medical Center, PO Box 9101 (960), 6500 HB Nijmegen, The Netherlands

## Abstract

**Background:**

The availability of validated laparoscopic simulators has not resulted in sustainable high-volume training. We investigated whether the validated laparoscopic serious game Underground would increase voluntary training by residents. We hypothesized that by removing intrinsic barriers and extrinsic barriers, residents would spend more time on voluntary training with Underground compared to voluntary training with traditional simulators.

**Methods:**

After 1 year, we compared amount of voluntary time spent on playing Underground to time spent on all other laparoscopic training modalities and to time spent on performing laparoscopic procedures in the OR for all surgical residents. These data were compared to resident’ time spent on laparoscopic activities over the prior year before the introduction of Underground.

**Results:**

From March 2016 until March 2017, 63 residents spent on average 20 min on voluntary serious gaming, 17 min on voluntary simulator training, 2 h and 44 min on mandatory laparoscopic training courses, and 14 h and 49 min on laparoscopic procedures in the OR. Voluntary activities represented 3% of laparoscopic training activities which was similar in the prior year wherein fifty residents spent on average 33 min on voluntary simulator training, 3 h and 28 min on mandatory laparoscopic training courses, and 11 h and 19 min on laparoscopic procedures.

**Conclusion:**

Serious gaming has not increased total voluntary training volume. Underground did not mitigate intrinsic and extrinsic barriers to voluntary training. Mandatory, scheduled training courses remain needed. Serious gaming is flexible and affordable and could be an important part of such training courses.

## Introduction

Most surgical departments have invested in creating simulation-based skills training facilities. This is caused by the need for residents to master an increasing number of highly complex surgical procedures which cannot be done in the operating room (OR) due to legal and ethical concerns regarding patient safety, resident workweek restrictions, and costs of OR time [1–3]. Laparoscopic simulators have been successfully validated and skills transfer to the operating room has been established [4–8]. The availability of validated laparoscopic simulators, however, has not resulted in sustainable, high-volume voluntary simulator training [9, 10]. This may be caused by intrinsic and/or extrinsic factors. Intrinsic (motivational) factors include perceived utility and whether or not the training activities are perceived as fun. Extrinsic factors include workweek restrictions, remote simulator locations, and the amount of mandatory training courses residents have to complete [11].

As to voluntary simulator training, the novel educational approach of serious gaming is well positioned to mitigate the various factors that are thought to limit the utilization of laparoscopic simulators. Serious games are designed to be entertaining and to provide an active learning environment for the player to develop professional skills [12–14]. Learning in serious games occurs by increasing intrinsic motivation through gameplay that engages learners in challenges that adapt to their growing in-game skills in a non-repetitive manner [15, 16]. In addition, extrinsic factors are addressed by lowering the barrier to training by facilitating training outside the skillslab on generic, widely disseminated digital platforms. Travel time to the skillslab is avoided, and the game can be played at short pauses during the workday or casually in an informal setting.

In earlier research, gaming elements such as competition and the option to train onsite (i.e., close to residents’ workspace) have been implemented in laparoscopic curricula. This led to a slight increase in resident participation rates. In these studies, however, single gaming elements were added to existing laparoscopic training technology [11, 17–19]. It remains in question whether a dedicated serious game can increase training volume by motivating residents to engage in voluntary training.

In this study we used the validated serious game *Underground* (developed for laparoscopic skills training) to investigate whether residents would show increased voluntary training [20–24]. We hypothesized that by removing intrinsic barriers (by providing a fun and challenging serious game) and extrinsic barriers (less travel time, use at work-pauses, casual use), residents would spend more time on voluntary training with Underground compared to voluntary training with traditional simulators.

## Material and methods

### Participants

In March 2016, the surgical residents working at the departments of general surgery, urology, and gynecology of the Radboud University Medical Center (Nijmegen, the Netherlands), were introduced to the serious game Underground through three scheduled introduction meetings, held at their respective departments. Residents in the control condition, working in the aforementioned departments in the prior year, did not have access to Underground and did not receive an introduction. No IRB approval was needed under Dutch law for this type of observational educational study.

### Study design

We installed the serious game Underground in the residents’ offices at the departments of general surgery, urology, and gynecology. All three groups of residents received an introduction in which they were explained how the system works, how to handle the controllers and how to troubleshoot if necessary. Underground was introduced as an addition to the traditional laparoscopic simulators and the use of the serious game or simulators was not mandatory. The simulators were located at our skillslab facility within the hospital building but not in the proximity of the ORs or normal wards. All residents were aware of its location and were already introduced to the available simulators. We collected laparoscopic training data between March 2016 and March 2017 for all surgical residents on all laparoscopic training modalities. Laparoscopic training modalities were: voluntary serious gaming (Underground), voluntary simulator training (FLS videotrainer, and LapSim Virtual Reality trainer) and mandatory laparoscopic training courses. Training times were compared to time spent on performing laparoscopic procedures in the OR. To contextualize these data, we collected laparoscopic training data for voluntary simulator training, mandatory laparoscopic training courses, and laparoscopic OR time from the period between March 2015 and March 2016 (a year before, when Underground was not yet introduced).

### Apparatus: hardware and serious game

**Fig. 1:**
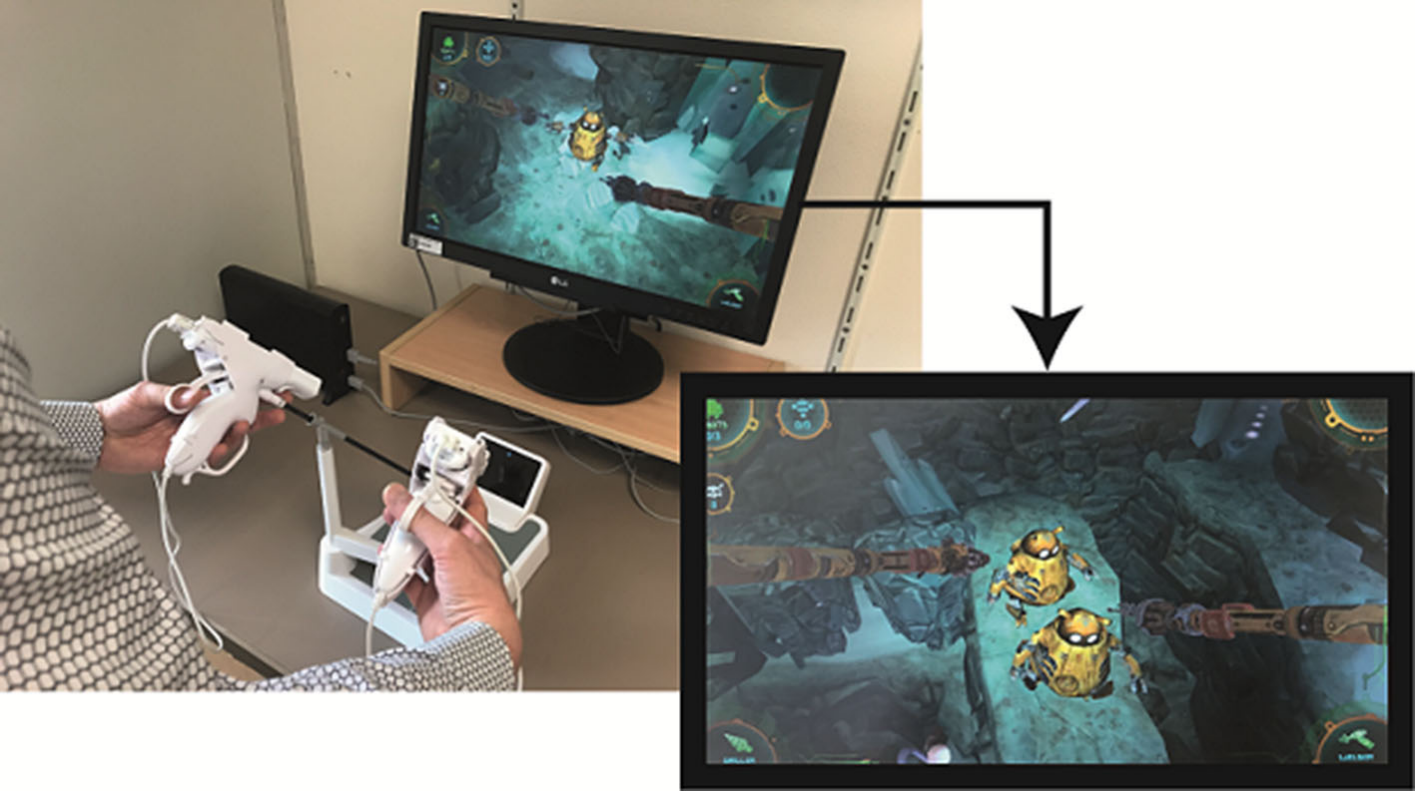
On the left the hardware interface of Underground. On the right two robots that have to be guided to the exit of a system of mineshafts by two probes inspired by laparoscopic instruments

Version 1.1 of Underground was used. The setup consisted of a Nintendo Wii U gaming console with remote controllers placed in a game-specific laparoscopic interface and a LG 21″ HD LCD screen [25] (Fig. 1). The sensitivity of the controllers was set at the highest possible level to limit choppy movements from the in-game instruments [26].

Within the serious game, players have to guide robots to the surface from an abandoned system of mineshafts while solving puzzles and creating routes by drilling, heating and grasping with their in-game robotic arms. These arms are controlled with the Nintendo Wii standard Nunchuk controllers, held by game-specific laparoscopic adapters. The movements needed for successful gameplay are based on the movements made during laparoscopic surgery. The main goals are learning to anticipate inverted movements by working over a fulcrum, eye-hand coordination, depth perception, and ambidexterity. When residents start the game for the first time, a tutorial begins in which the game mechanics are explained. The game has four distinct themes, each with five levels. After completing these four themes, the game culminates in a final ‘boss’ level [27]. The gameplay becomes more difficult as the trainee completes more levels by introducing enemies, obstacles, necessitating increasingly complex instrument movements. Validity criteria for Underground have been met in previous research; however, we are not aware of research formally matching Underground difficulty levels to current laparoscopic simulators [21, 22, 28]. As Underground is aimed at basic skills training and not specific laparoscopic procedures, we expect the greatest utility to be in early phase residency training.

### Data preparation

#### Training volume serious game underground

Multiple accounts can be created on a Nintendo Wii U to represent individual players. Per account, the number of gaming sessions is recorded, as well as time played per session. These data were collected for all accounts for the period of the study, to calculate the total time spent on Underground for all three resident offices’ Underground installations.

#### Training volume various simulators in the skillslab

Prior to laparoscopic simulator training, residents make a reservation for the skillslab facility through a digital calendar (to make sure the facility is not double booked). We used the reserved time slots to calculate voluntary time spent on laparoscopic simulators by residents.

#### Training volume mandatory laparoscopic training courses

In each departments’ training plan for residents the amount of time that has to be spent on mandatory training courses for each postgraduate year in training (PGY) is described. We calculated the total amount of training hours by multiplying the number of residents for each PGY by the number of hours described in the training plans.

#### Time spent on laparoscopy in the operating room

We collected the amount of time registered for each laparoscopic procedure performed by residents as either primary, secondary or tertiary surgeon from the hospital information system to calculate the total time spent on laparoscopic procedures by residents.

### Data analysis

Data were analyzed using the software package IBM SPPS Statistics for Windows, version 25. Resident training times for each training modality and resident time spent on laparoscopic procedures in the OR are reported with descriptive statistics.

## Results

### Participants

Sixty-three residents, distributed over Surgery, Urology and Gynecology, trained at Radboudumc from March 2016 until March 2017, and this number was 50 during the prior year (Table 1). Due to the rotation schedule for residents, most of them spent only a part of this period at Radboudumc. At any given moment, on average forty residents were part of the Radboudumc training program. More residents in their specialization phase (PGY 5&6) were in training over 2016–2017.

**Table 1:** Residents in training during March 2016–March 2017

	Surgery		Urology		Gynecology	
	2015–2016	2016–2017	2015–2016	2016–2017	2015–2016	2016–2017
*PGY*						
1	2	2	1	0	0	0
2	2	1	0	0	0	0
3	3	2	1	5	11	12
4	8	12	4	2	11	14
5	5	4	2	0	0	2
6	0	0	0	4	0	3
Total unique residents per year (*n*)	20	21	8	11	22	31

### Voluntary training

**Fig. 2:**
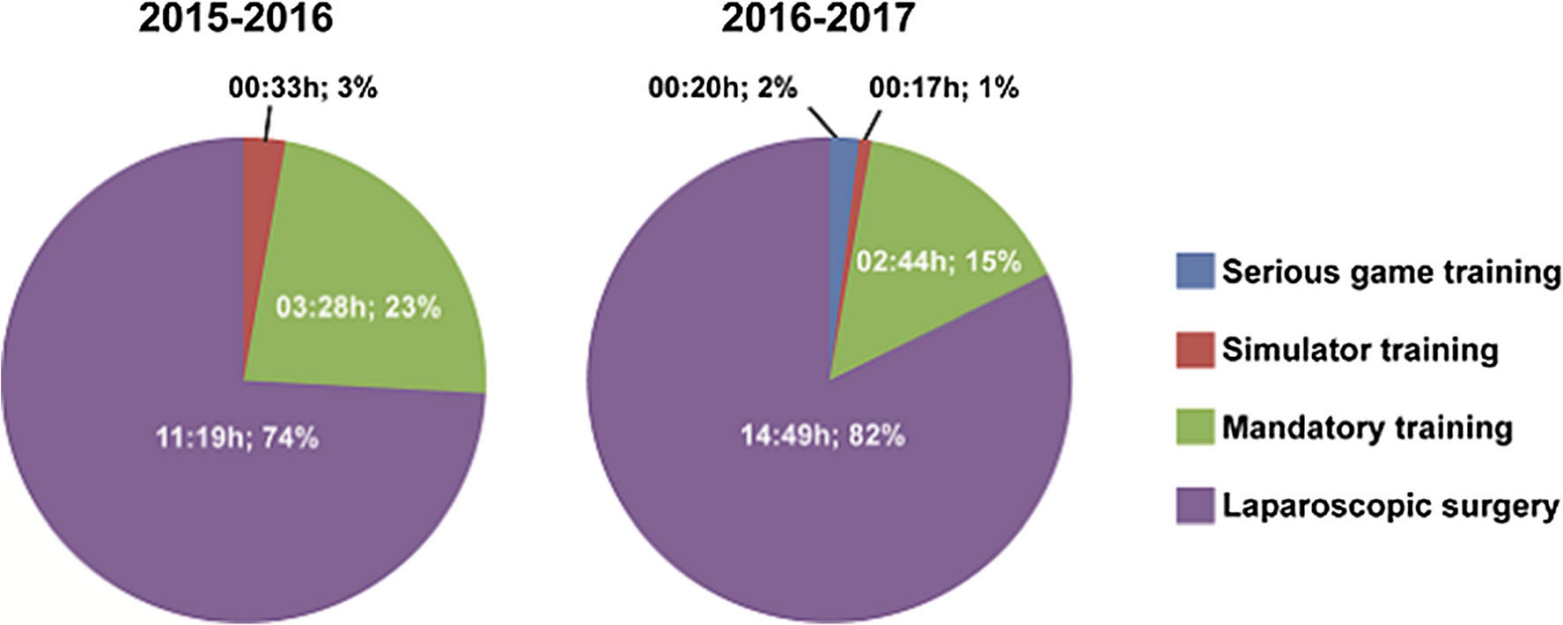
Averaged, per-resident training time distribution over the available laparoscopic training modalities and procedures from March 2015 to March 2016 and from March 2016 to March 2017 (hours/minutes, percentage of total time spent on laparoscopy)

In the period from March 2016 until March 2017, residents spent on average 2% (20 min) of their time on serious gaming, 1% (17 min) on voluntary simulator training, 15% (2 h and 44 min) on mandatory laparoscopic training courses, and 82% of their time (14 h and 49 min) on laparoscopic procedures. From March 2015 until March 2016, residents spent on average 3% (33 min) on voluntary simulator training, 23% (3 h and 28 min) on mandatory laparoscopic training courses, and 74% of their time (11 h and 19 min) on laparoscopic procedures, Fig. 2.

## Discussion

To our best knowledge, this is the first study evaluating voluntary psychomotor skills training with a serious game. In contrast to the common opinion that residents would spent more time on voluntary training if only training were more fun and accessible [29, 30], we found that the ready availability of the serious game Underground did not result in an increase in voluntary training time. Overall, time spent on voluntary training was marginal compared to time spent on mandatory laparoscopic training courses and compared to time spent on laparoscopic procedures. This low utilization rate of laparoscopic training modalities is in line with results reported for traditional voluntary laparoscopic simulator training [11]. Lowering the extrinsic barriers of remote training locations and scheduling issues [9, 18, 19] was not sufficient to structurally increase the voluntary training effort of residents in this study.

As to the intrinsic factor of fun, gaming elements present in Underground such as a story mode, a scoring system, game levels of increasing difficulty, and a bonus system (all of which are known to motivate players by providing a sense of competence and/or presence [12, 26, 31–36]) did not result in an increase in training volume. Other gaming elements such as competition and multiplayer functionality however are not incorporated in Underground software. In several observational studies, Introducing a simple element of competition into current laparoscopic curricula led to an increased participation rate of up to 30% [17, 18].

In light of this, the addition of an element of competition to Underground might therefore have improved voluntary participation rates. However, we do not expect this improvement to be substantial because published face validity data as well as our unpublished face validity data show that residents enjoy playing Underground and strongly like the concept of serious gaming. Also, they perceive Underground as a valuable approach to laparoscopic skills training [26, 37].

Another, more likely explanation for the low volume of voluntary laparoscopic training activities might be the disincentive for voluntary simulator training caused by allowing residents to practice on actual patients after a mandatory basic laparoscopy introduction course. This is supported by reports that residents feel restricted in time and bound to clinical responsibilities and they might therefore consider doing laparoscopy in the OR as training in practice [10, 18, 19, 29, 30]. In addition, studies have shown that even if simulation training is mandatory, utilization rates are low which may suggest that training in the OR is given higher priority [9, 17, 38]. Thus, it seems that mandatory, scheduled training courses remain needed. Analogous to the situation in the airline industry, it might be worthwhile to consider making access to laparoscopic procedures conditional on prior certification.

Each year in training comes with different learning goals and activities for that year. This explains why there is a difference in time spent on mandatory training and laparoscopic surgery over both years as the number of residents per PGY were not the same. Compared to 2015 residents spent less time on voluntary simulator training. Residents may have divided their available time over Underground and simulator training rather than spending more time on laparoscopic training which is in line with the perceived feeling of being restricted in time and bounded to clinical work. Considering Underground as a voluntary training opportunity, residents spent roughly as much time on voluntary training over both 2015 and 2016. Regardless of PGY, residents do not prioritize voluntary training.

## Limitations

The results of this study reflect the situation in one Dutch university medical center and may not be generalizable to other hospitals inside and outside the Netherlands. However, informal conversations in a variety of setting, including international conferences, lead us to believe that our results reflect the typical situation with regards to laparoscopic training.

Limited voluntary gameplay may in part have been caused by technical issues, which were reported by 30% of our residents in response to a face validity questionnaire on Underground. This is in line with the study of Overtoom et al., who suggest these issues need to be addressed prior to adaptation in the skillslab [26]. However, the majority of our residents did not report such issues, and we therefore do not expect technical issues to be the cause of the low utilization rate.

## Conclusion

Using serious gaming to remove intrinsic and extrinsic barriers to voluntary laparoscopic skills training for residents did not result in an increase in voluntary training volume. Voluntary training is a minor part of the total volume of the laparoscopic activities of residents. Mandatory, competency-driven training courses remain needed to increase training volume. Residents of the surgical specialties perceive serious gaming as fun and useful, and serious gaming can be an important part of such training courses.
